# The Future of Blood Testing Is the Immunome

**DOI:** 10.3389/fimmu.2021.626793

**Published:** 2021-03-15

**Authors:** Ramy A. Arnaout, Eline T. Luning Prak, Nicholas Schwab, Florian Rubelt, the Adaptive Immune Receptor Repertoire Community, Ramy A. Arnaout

**Affiliations:** ^1^Department of Pathology and Division of Clinical Informatics, Department of Medicine, Beth Israel Deaconess Medical Center, Boston, MA, United States; ^2^Department of Pathology, Harvard Medical School, Boston, MA, United States; ^3^Department of Pathology and Laboratory Medicine, Perelman School of Medicine, University of Pennsylvania, Philadelphia, PA, United States; ^4^Department of Neurology and Institute of Translational Neurology, University of Muenster, Muenster, Germany; ^5^Roche Sequencing Solutions, Pleasanton, CA, United States

**Keywords:** adaptive immune receptor repertoire (AIRR), diagnostic test, T-cell receptor repertoire, antibody repertoire, analyses, immunome, immunomics, clinical laboratory testing

## Abstract

It is increasingly clear that an extraordinarily diverse range of clinically important conditions—including infections, vaccinations, autoimmune diseases, transplants, transfusion reactions, aging, and cancers—leave telltale signatures in the millions of V(D)J-rearranged antibody and T cell receptor [TR per the Human Genome Organization (HUGO) nomenclature but more commonly known as TCR] genes collectively expressed by a person’s B cells (antibodies) and T cells. We refer to these as the *immunome*. Because of its diversity and complexity, the immunome provides singular opportunities for advancing personalized medicine by serving as the substrate for a highly multiplexed, near-universal blood test. Here we discuss some of these opportunities, the current state of immunome-based diagnostics, and highlight some of the challenges involved. We conclude with a call to clinicians, researchers, and others to join efforts with the Adaptive Immune Receptor Repertoire Community (AIRR-C) to realize the diagnostic potential of the immunome.

## Introduction

**Figure 1:**
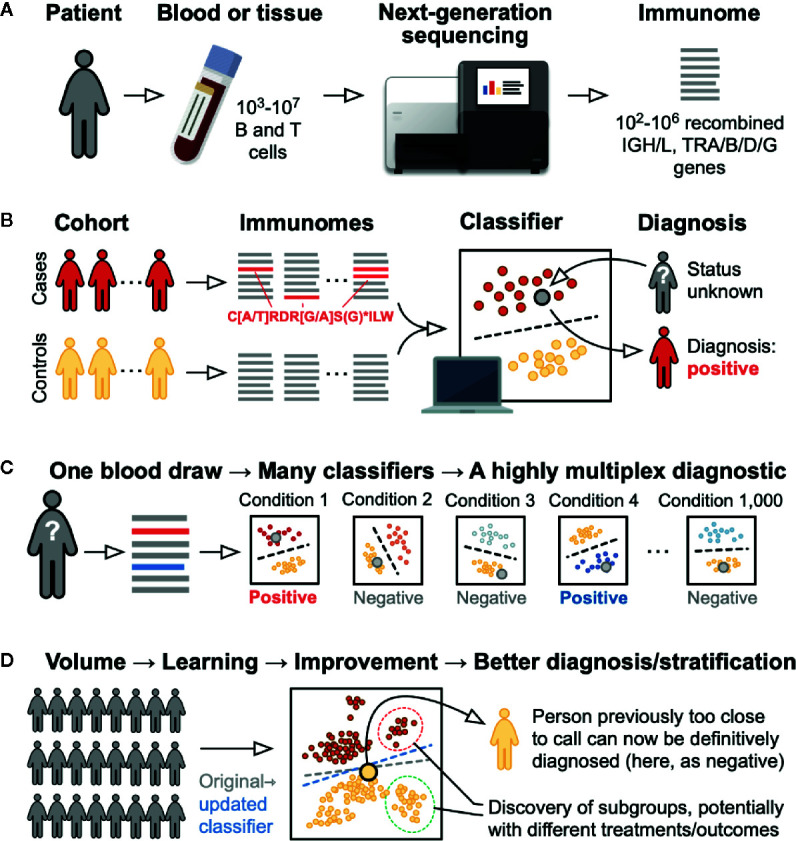
Immunome-based diagnostic testing. **(A)** Testing begins with a standard clinical blood draw. The recombined immunoglobulin (B cell receptor) and TR (T cell receptor) rearranged genes are sequenced, leading to a list of the hundreds of thousands of different sequences present in the sample: i.e., the patient’s immunome. **(B)** To develop a test for a specific condition, immunomes are sequenced from a set of cases positive for the condition and an appropriately matched set of controls. Robust statistical and mathematical techniques are used to identify patterns in the form of specific sequences, motifs (e.g., the IGH CDR3 shown in red), and clusters, as well as changes in overall sequence diversity, that are characteristic of the cases but not the controls. Based on these and other sequence features, and with the help of computational techniques, a classifier is developed that reliably separates the two groups. Using this classifier, a patient of unknown status (large gray circle) can be diagnosed by sequencing that patient’s immunome and looking for presence or absence of the pattern. **(C)** By applying classifiers for many different conditions to the sequence from a single blood draw, many different conditions can be diagnosed simultaneously, yielding a highly multiplexed diagnostic assay. **(D)** As more individuals are tested for a specific condition, the classifier for that condition will be refined—in AI terms, it “learns”—allowing individuals who were previously unclassifiable to be diagnosed and potentially allowing stratification of patients who might benefit from different treatments or who might have a different prognosis or risk of disease development.

The convergence of high-throughput sequencing technologies with advances in computation and data science has given sequencing a growing role in clinical diagnosis. Examples of high-throughput sequencing applications that have begun to enter the clinic in recent years include cancer-gene sequencing to identify clinically actionable mutations and whole-genome and metagenomic sequencing to resolve medical mysteries (1–4). The main appeal of sequencing as a diagnostic modality is its potential to detect all of the possible variants of a given gene or genes in a single test. In situations where there are many variants that may be diagnostically or prognostically useful, as is the case for cancer and in genetic disorders such as cystic fibrosis, sequencing has been shown to be more sensitive than tests that target a limited set of variants, for example using PCR (5, 6). NGS-based testing has also advanced the field of immunogenomics, providing a more streamlined means of identifying and cataloguing novel human leukocyte antigen (HLA) genes and associating allelic variants and haplotypes with diseases and immune perturbations (see below). Because of their intimate association with many different diseases, B- and T-cell *immunomes* will feature prominently in the future of clinical lab testing (7) (**Figure 1**).

The term “immunome” refers to the repertoire of a person’s antibodies and TRs, most often measured from the blood, which contains roughly 50,000–440,000 B cells and 600,000–3,500,000 T cells per ml in a healthy adult (8–10). Antibodies and TRs are encoded by genes of extraordinary diversity: each person’s immunome contains millions of distinct rearranged antibody and TR genes (henceforth simply “genes”) (11–14). This diversity is what makes it possible for an individual’s immune system to recognize and respond to different antigens in vaccination, infection, autoimmunity, cancer, and other conditions. The binding of an antibody expressed on the B-cell surface to one of its specific antigens—for example, influenza hemagglutinin or the spike protein of SARS-CoV-2—can promote B cell activation, division, and differentiation, resulting in the production of antibodies. For alpha-beta TRs on the surface of T cells, the antigen is typically a peptide that is presented to the TR in the context of the major histocompatibility complex (gamma-delta T cells do not necessarily require MHC).

## The Immunome as Diagnostic

For diagnostic purposes, the expansion of antigen-specific B or T cell clones acts as a signal amplifier, indicating a response to a specific antigen or antigens in the form of an increased frequency of cells expressing antigen-specific antibody and/or TR genes in the immunome. Such increases can now be measured quantitatively through high-throughput sequencing in an application known as adaptive immune receptor repertoire sequencing, or AIRR-seq. In principle, repeated cycles of antigen encounter, clonal expansion, and repertoire diversification result in a personalized record of a patient’s immune status across vaccination, infection, autoimmunity, transplant rejection, transfusion reactions, and cancer. AIRR-seq makes it possible to read this record. The past few years have seen an explosion of proofs of principle in the research literature. For example, in patients who have had influenza or received an influenza vaccine, AIRR-seq has demonstrated an increase in influenza-specific antibody and TR genes in the blood and in tissues (15–20). Similar results have been demonstrated in viral infections as diverse as dengue and SARS-CoV-2 (21–28). Noteworthy in this regard is the current effort to discern a T cell fingerprint for SARS-CoV-2 exposure, immune status and possibly even immunopathology in the ImmuneCODE project, a collaboration between Adaptive Biotechnologies and Microsoft, which leverages a rapidly growing and publicly accessible dataset of over 1,400 TR immunomes from individuals who were exposed to SARS-CoV-2 (28, 29). Patterns have been reported in a host of autoimmune diseases such as lupus (30–33), and antibodies and TRs against neoantigens have been reported across solid tumors and in specific cancers such as melanoma (34). These examples and many others increasingly support the view that disease-specific patterns in immunomes are widespread and are likely to be clinically useful.

Immune repertoire profiling is already in clinical use for the diagnosis and monitoring of hematologic malignancies, most frequently of the B cell lineage. The hypervariable third complementarity determining region of the antibody heavy chain or TR beta-chain genes can be used as a clonal fingerprint. Identifying expansions of the same CDR3 sequence and tracking these expansions over time can be used to monitor the frequency of a specific B cell or T cell clone and thereby test for minimal residual disease in these conditions (35). Furthermore, the presence of somatic hypermutations in the antibody variable gene sequence can be used as a prognostic marker in chronic lymphocytic leukemia (36). In addition, patterns within the immunome have been shown to correlate with likelihood of disease or treatment response in several clinical contexts. For example, limited diversity of the repertoire has been associated with frailty during aging (37, 38) and provides evidence of immunodeficient states that are caused by disease or therapies such as bone marrow transplantation. Skewing of immunomes towards overrepresentation of particular variable region genes, CDR3 sequences, or other more complex motifs may serve as fingerprints for disease or disease susceptibility, and studies of immunologic exposure and motifs may be useful for monitoring of immune responses to pathogens as well as therapeutic vaccines (32, 33, 39–41). Immunome profiling thus provides an opportunity to mine immune systems at the individual and population levels to identify signatures that, combined, would represent a “universal” diagnostic laboratory test. Such a clinical test would represent a natural next step for investigations into the role of adaptive immunity in clinical laboratories.

## Advantages of AIRR-SEQ

AIRR-seq provides a global view of the immune system in a single snapshot that can be interpreted with different computational methods. Recent progress has already brought the cost and turnaround time of AIRR-seq tests within range of other clinical NGS tests, especially those that must be performed at reference laboratories, also known as “send-out tests” (as opposed to tests that can be performed on site at the hospital). Most critically, sequencing has the fundamental advantage of being an unbiased method, which can in principle reveal atypical immune responses, or responses to new or emerging pathogens such as SARS-CoV-2 without the cost and delay required to develop new pathogen-specific reagents (42–44). Regardless of the condition, the sequencing procedure is the same; what differs is the computational query for each test result: one for influenza, another for lupus, and so on. Each query parses the data for immunoglobulin or TR sequences, motifs, or clusters specific for the given condition. In this manner, AIRR-seq testing can reveal unusual or unexpected patterns in a series of patients with a shared condition. Immunomes and specific sequences can be made easily available through existing resources that have been developed in part in anticipation of diagnostic purposes; these resources include the AIRR Data Commons, which can be accessed *via* the iReceptor Plus portal (45) and using the AIRR-C API (46). Reference sequences for specific variable genes, alleles and haplotypes can be found at IMGT (47), the Open Germline Reference Database (OGRDB) (48), and the VDJdb database (49) and antigen-specific antibodies and TRs can be queried in the Immune Epitope Database (IEDB) using a suite of tools (50).

Immunome-based diagnostics also align well with the vision of so-called learning health systems, in which the experience of each patient and physician contribute to an improved understanding of disease prevention and management for the benefit of all (51–57). With enhanced data collection in the electronic medical record, secure data sharing, and advances in machine learning, this vision is gradually moving closer to becoming a reality. Just as the growth of repositories such as the UK Biobank increases the power for finding patterns across many conditions, each newly sequenced immunome adds statistical power for finding immunome patterns across the broader population. Data from AIRR-seq based tests could be used for continuing improvement of their diagnostic power. Indeed, efforts by the growing Adaptive Immune Receptor Repertoire Community (AIRR-C; www.antibodysociety.org/the-airr-community/) (58) and by EuroClonality (www.euroclonality.org/) have already led to the development of several high-quality curated public, searchable databases of sequences and their specificities, as well as standards for repertoire data (58–60).

## Challenges and Opportunities

There are of course several challenges to address for immunome-based blood testing to become a practical and useful clinical reality, just as there were (and are) for incorporating cancer sequencing, whole-genome sequencing, and metagenomics into patient care. First, the timing of sample collection is important to consider, particularly in investigations of infections or vaccine responses. Recall responses can arise within days of an exposure, but initial responses may take a week or longer to become detectable; durations of responses vary from months or less to lifelong. These considerations mean that immunome-based testing may eventually play multiple different roles for screening, diagnosis, and rule-out testing, depending on the condition and durability of the immune response. Second, protocols for sample preparation will have to be standardized and validated. These will need to cover cases in which sequencing will be performed from whole blood or peripheral blood mononuclear cells (PBMCs) *vs.* select subsets (*e.g.* memory B cells, effector T cells); whether single IGH, IGK/L, TRA, TRB, TRG, TRD chains or paired chains will be sequenced (the latter is currently lower throughput and more expensive and requires intact cells); which regions of each chain are sequenced; whether the sequencing is from genomic DNA (agnostic to the cell subset or activation state), mRNA (potentially including isotype information but also may over-represent activated cells), or both; and so on. Third, computational pipelines will likewise need to be clearly described and validated, from sequence assembly (where necessary), to sequence annotation, to correction for sampling and sequencing errors, to the statistical and/or machine learning methods used. All of the stages of this process will need to be rigorously validated to achieve regulatory approval. Fourth, sequencing and computational resources will have to be in place to guarantee clinically viable price points and turnaround times. Fifth, samples will have to be clinically annotated, and this and indeed every process will have to be carefully vetted for adherence to patient privacy directives and related legislation. Sixth, systems for storing and sharing the new clinical data must be set up, to avoid silos and instead maximize the statistical power of widespread testing (as in the vision for learning health systems). And seventh, the signatures, signals, and patterns on which the tests are based must be robust and interpretable, which will likely require the development of “reference ranges” that are relatable to age, gender, genetic background (*e.g.* ethnicity, gene-segment variants, MHC dependency), and geography (for environmental or infectious exposures).

Fortunately, none of these issues is unique to the immunome; all are or have been regularly encountered in clinical pathology/laboratory medicine, and especially in the development of existing high-throughput sequence-based tests. Moreover, these issues have long been at the center of discussions by the AIRR-C and others to develop guidelines to facilitate eventual clinical adoption (58, 59, 61). For the first time, AIRR-seq offers the possibility of a universal laboratory test that potentially addresses many day-to-day issues in clinical practice: diagnosis and disease prognosis, resistance or response to drugs, immunodeficiency, anti-tumor and immunotherapy responses, and monitoring for progression vs. recovery. In addition, it introduces bioinformatics as the key testing component, suitable for the current era of personalized immuno-medicine, data science, and data-driven patient care. For AIRR-seq based clinical testing to reach its full potential, broad implementation will be required. Similar excitement and partnership have already led to the development of sequencing-based tests in cancer and inherited disease, and is also occurring for the microbiome. We hope that clinicians, laboratorians, researchers, and funding organizations will join efforts to further realize the diagnostic potential of the immunome to help patients with a wide variety of important health conditions.

## Data Availability Statement

The original contributions presented in the study are included in the article/**Supplementary Material**. Further inquiries can be directed to the corresponding authors.

## Author Contributions

RA, FR, NS, ELP, and the AIRR-C conceived of the work. RA wrote the initial drafts and prepared the figure. RA, FR, NS, ELP, and the AIRR-C edited the final draft. All authors contributed to the article and approved the submitted version.

## Conflict of Interest

Author FR was employed by Roche Sequencing Solutions.

The remaining authors declare that the research was conducted in the absence of any commercial or financial relationships that could be construed as a potential conflict of interest.
