# Supplementary material for: The Future of Blood Testing Is the Immunome
Source: Front Immunol. 2021 Mar 15;12:626793. doi: 10.3389/fimmu.2021.626793 (PMC8005722; doi:10.3389/fimmu.2021.626793)
Supplement: Supplementary file 1 [file DataSheet_1.docx]

**The Future of Blood Testing Is the Immunome:
Supplementary Information**

**Full author list**

Ramy A. Arnaout, MD, DPhil, Department of Pathology and Division of Clinical Informatics, Department of Medicine, Beth Israel Deaconess Medical Center, Boston, MA 02215 USA and Department of Pathology, Harvard Medical School, Boston, MA 02115 USA

Rohit Arora, PhD, Beth Israel Deaconess Medical Center, Boston, MA 02215 USA. Current address: Iktos, 75017 Paris, France

Rachael Bashford-Rogers, MChem, PhD, University of Oxford, Wellcome Centre for Human Genetics, Oxford OX3 7BN, Oxfordshire, UK

Felix Breden, PhD, Department of Biological Sciences, Simon Fraser University, Burnaby, BC, Canada

Syed Ahmad Chan Bukhari, PhD, St. Johns's University, Jamaica, NY 11439 USA

Brian Corrie, PhD, Simon Fraser University, Burnaby, BC, Canada

Lindsay G. Cowell, PhD, UT Southwestern Medical Center, Dallas, TX 75390 USA

Sol Efroni, PhD, Bar-Ilan University, Faculty of Life Sciences, Bar-Ilan University, Ramat Gan, Israel

Christopher Gooley, Microsoft Research, Redmond, WA 98052 USA

Victor Greiff, PhD, University of Oslo, Rikshospitalet, Oslo, Norway

Jason Vander Heiden, PhD, Department of Bioinformatics and Computational Biology, Genentech, South San Francisco, CA 94080 USA

Yoshinobu Koguchi, MD, PhD, Providence Cancer Institute, Portland, OR 97213 USA

Ton Langerak, PhD, Erasmus MC, University Medical Center, Rotterdam, The Netherlands

Theam Soon Lim, PhD, Institute for Research in Molecular Medicine, Universiti Sains Malaysia, 11800 Penang, Malaysia

Eline Luning Prak, MD, PhD, Department of Pathology and Laboratory Medicine, Perelman School of Medicine, University of Pennsylvania, Philadelphia, PA 19104 USA

Encarnita Mariotti-Ferrandiz, PhD, Sorbonne Université, U959, Immunology-Immunopathology-Immunotherapy (i3), Paris, France

Susanna Marquez, Yale University New Haven, CT 06511 USA

Pieter Meysman, PhD, University of Antwerp, Antwerp, Belgium

Enkelejda Miho, PhD, Institute of Medical Engineering and Medical Informatics, School of Life Sciences, FHNW University of Applied Sciences and Arts Northwestern Switzerland, Muttenz, Switzerland; 2SIB Swiss Institute of Bioinformatics, Lausanne, Switzerland; 3aiNET GmbH, Basel, Switzerland

Keshav Motwani, Department of Pathology, Immunology, and Laboratory Medicine, University of Florida Diabetes Institute, Gainesville, Florida, USA

Nima Nouri, PhD, Yale, New Haven, CT 06511 USA

Milena Pavlović, MSc, University of Oslo, Blindern, Oslo, Norway

Florian Rubelt, PhD, Roche Sequencing Solutions, Pleasanton, 94588 CA USA

Geir Kjetil Sandve, PhD, University of Oslo, Blindern, Oslo, Norway

Nicholas Schwab, PhD, Department of Neurology and Institute of Translational Neurology, University of Muenster, 48149 Muenster, Germany

Igor Snapkov, MD, PhD, Department of Immunology, University of Oslo, Oslo, Norway

Cinque Soto, PhD, Vanderbilt Vaccine Center, 11475 MRBIV, 2213 Garland Avenue, Nashville, TN 37232, USA; Department of Pediatrics, Vanderbilt University Medical Center, Nashville, TN 37232 USA

Ulrik Stervbo, PhD, Center for Translational Medicine, Immunology, and Transplantation, Marien Hospital Herne, University Hospital of Ruhr-University Bochum, Germany

Johannes Trück, MD, DPhil, University Children's Hospital Zurich, Zurich, Switzerland

Henk-Jan van den Ham, PhD, ENPICOM B.V. ‘s-Hertogenbosch, The Netherlands

Corey Watson, PhD, Department of Biochemistry and Molecular Genetics, University of Louisville School of Medicine, Louisville KY 40202 USA

Cédric R. Weber, Department of Biosystems Science and Engineering, ETH Zürich, 4058 Basel, Switzerland
